# Measurement and Characterization of Rotational Errors of Aerostatic Bearings in Subnanometer Accuracy

**DOI:** 10.3390/mi14101952

**Published:** 2023-10-19

**Authors:** Ping Wang, Lingbao Kong, Huijun An, Minge Gao, Hailong Cui, Dajiang Lei

**Affiliations:** 1 Shanghai Engineering Research Centre of Ultra-Precision Optical Manufacturing, Fudan University, Shanghai 200438, China; 19110720017@fudan.edu.cn (P.W.); 20110720010@fudan.edu.cn (H.A.); 19110860002@fudan.edu.cn (M.G.); 2 Sichuan Precision and Ultra-Precision Machining Engineering Technology Center, Chengdu 610200, China; cuihailong61@foxmail.com (H.C.); leidajiang@163.com (D.L.)

**Keywords:** measurement, atomic force microscopy (AFM), rotational errors, aerostatic bearings

## Abstract

Measuring the running accuracy of aerostatic bearings is challenging because of the high-precision requirements in rotational motion. This paper presents an ultra-high precision measurement method for aerostatic bearings using atomic force microscopy (AFM) as the displacement sensor. The Donaldson reversal method was used to separate the artifact form errors from the measurement results. A measurement system was developed with the integration of an AFM module. The effects of sensor nonlinearity, environmental noise, and structural vibration on the measurement results were effectively suppressed in the system. The experimental results show that the measurement achieves up to subnanometer accuracy.

## 1. Introduction

Aerostatic bearings have been widely used in ultra-precision machine tools [1]. The motion errors of aerostatic bearings greatly influence the machining accuracy of optical components [2,3]. Research on the rotation accuracy of aerostatic bearing mainly includes theoretical modelling and measurement methods. For instance, Cappa et al. [4] theoretically investigated the effect of different factors on the radial motion error of an aerostatic journal bearing using a steady-state model. Although the rotation errors of aerostatic bearing can be predicted theoretically, there is still a large deviation between the predicted results and actual data. This is because the measurement results of rotation accuracy are affected by many factors, such as component and installation accuracy, and error separation approaches [5,6,7]. Thus, methods and systems for measuring the rotation errors of aerostatic bearings with high accuracy must be developed.

Over the last few years, to validate the theoretical studies, researchers have proposed numerous measuring systems and error separation methods to improve the measurement accuracy of rotation errors of aerostatic bearings [8,9,10]. For instance, Haitjema [11] proposed a combination method to improve error separation accuracy and reduce measurement steps from the standard 12 steps to 7 steps, with which uncertainty could be achieved in 4 nm. Meanwhile, Grejda et al. [12] proposed an improvement method that applied a high-precision indexing table and a reversal chuck to relocate both the sensor and artifact, which achieved subnanometer repeatability. Marsh et al. [13] compared reversal and multipoint error separation techniques, and proved that the rotation error of aerostatic bearings and artifact form error can be measured in subnanometer repeatability by both reversal and multipoint techniques. Cappa et al. [14] compared the Donaldson reversal, Grejda reversal, and multiprobe techniques and studied the effects of the positioning errors of artifacts and sensors, the positioning of the indexing table, the tilt error of the sensor, sensor misalignment, and artifact eccentricity on the measurement uncertainty. Tagne et al. [15] also introduced a Fourier-based method for identifying spindle error motion that effectively separates roundness deviations from spindle error motions with a reduced number of angular shifts. Wang et al. [16] analyzed measurement errors and their influence in the context of the combined measurement system. To separate error signals, they utilized the multi-step method, as well as Fourier transforms and inverse Fourier transform techniques. He et al. [17] utilize a composite laser target attached to the air spindle to mark its position during rotation, which enables the measurement of both the position and angle of the composite laser target, thereby obtaining spindle error data encompassing axial, radial, and tilt errors across five degrees of freedom. Pannackal et al. [18] introduced an approach that utilizes the Donaldson reversal technique with multiple probes, which eliminates the need for manual rotation and effectively separates test bar roundness errors from external random errors, reducing systematic effects. Marsh et al. [19] also compared three different error motion separation methods at the nanometer level, the Donaldson reversal, multiprobe, and multistep techniques, and confirmed that both synchronous and asynchronous errors should be considered for the realization of nanometric measurement accuracy. Synchronous errors represent the extent of identified rotational errors in aerostatic bearings and are widely utilized as critical indicators for characterizing aerostatic bearing performance. Conversely, asynchronous errors signify the magnitude of random rotational errors, with their manifestation strongly influenced by sensor performance and environmental conditions. However, the existing literature primarily focuses on error separation techniques for synchronous measurements, with limited research addressing the reduction in asynchronous measurement errors.

Because the rotation error of aerostatic bearings is directly measured by the displacement sensor, the measurement precision is significantly influenced by the resolution and nonlinearity of the displacement sensor, especially in asynchronous measurement results. Some high-precision non-contact capacitive displacement sensors can realize subnanometer resolution; however, their accuracy is often influenced by electromagnetic field and also needs a regular calibration [20,21]. Given their extremely high resolution and accuracy, atomic force microscopes (AFMs) have been widely used for nanometer-scale measurements [22], providing an enabling solution for achieving the subnanometer accuracy of asynchronous measurements. However, most commercial AFMs are limited to certain types and sizes of samples. Therefore, applying multistep, multipoint, or reversal methods to separate the artifact form error from the measured results using AFMs is very difficult. Furthermore, the measurement accuracy of AFMs is also affected by the microvibration of aerostatic bearings.

This paper concentrates on the design and implementation of an ultra-high precision measurement system for measuring the operational accuracy of aerostatic bearings at the subnanometer level. Based on the Donaldson reversal method, the measuring system was established through the integration of an AFM scanning module, together with the adjustment of modules, the optical microscope, and guideways. The effects of the resolution and nonlinearity of displacement sensors, environmental noise, and structure vibration on the measurement results were greatly suppressed by the designed system structures. A series of experimental studies for measuring aerostatic bearings was conducted to validate the precision and accuracy of the developed system.

## 2. Error Separation and Eccentricity Removal

Before the development of the measurement system, two important algorithms were first explained: the error separation method and the removal of the eccentricity of the arti-fact.

### 2.1. Error Separation Method

The most common error separation methods for separating the artifact form error from the measurement results are the multistep method, multipoint method, and reversal method [19]. However, the separation precision of the multistep method was significantly influenced by the number of steps and sampling points [5], and the reinstallation of displacement sensors also resulted in low efficiency and low precision [11]. Meanwhile, the multipoint method (three-point method) is more efficient as it only requires a single step for measurements. However, this approach faces significant challenges, including issues with harmonic suppression and uneven sensor sensitivities, leading to a decline in measurement accuracy [6].

**Figure 1:**
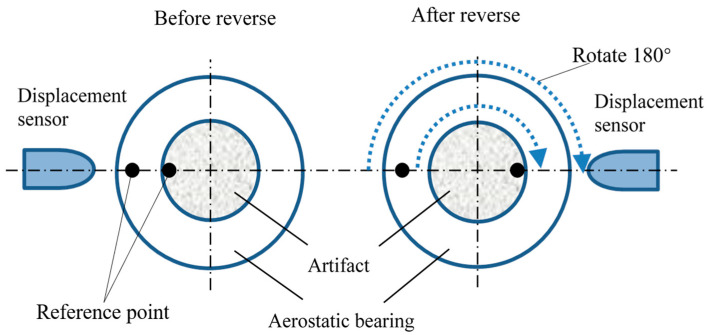
Principle of the Donaldson reversal method.

The reversal method is well known to be a perfect way to realize complete separation in theory. In this paper, the Donaldson reversal method was applied to separate artifact form errors from the measurement results. The principle of the Donaldson reversal method is shown in Figure 1. The artifact of a standard ball was installed at the end of the aerostatic bearing. Both the artifact and sensor were required to rotate precisely 180° after one measurement. The distance between the artifact and the sensor was recorded by the displacement sensor. The measurement data included the form error of the artifact and the rotation error of the aerostatic bearing. The relationship between these two errors can be expressed as follows:

(1) $$\left\{\begin{array}{l} T\left(\theta_{i}\right)=S\left(\theta_{i}\right)+R\left(\theta_{i}\right)\\ T^{\prime }\left(\theta_{i}\right)=-S\left(\theta_{i}\right)+R\left(\theta_{i}\right) \end{array}\right.$$

where $T\left(\theta_{i}\right)$ is the measurement data before reversal, $T^{\prime }\left(\theta_{i}\right)$ is the measurement data after reversal, $S\left(\theta_{i}\right)$ is the form error of the standard ball, and $R\left(\theta_{i}\right)$ is the radial rotation error of aerostatic bearing. Then, the rotation error of the aerostatic bearing was separated from the artifact form error by the following equation:

(2) $$\left\{\begin{array}{c} R\left(\theta_{i}\right)=\left(T\left(\theta_{i}\right)+T^{\prime }\left(\theta_{i}\right)\right)/2\\ S\left(\theta_{i}\right)=\left(T\left(\theta_{i}\right)-T^{\prime }\left(\theta_{i}\right)\right)/2 \end{array}\right.$$

### 2.2. Removal of the Eccentricity of the Artifact

**Figure 2:**
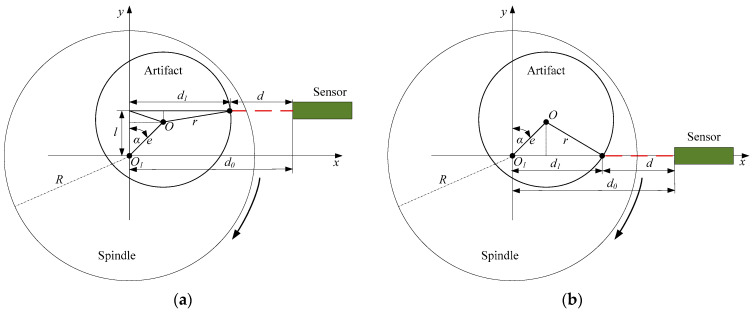
Schematic of artifact eccentricity effect: sensor with (**a**) and (**b**) without position deviation.

Besides the error separation, another important algorithm is the removal of the eccentricity of the artifact. The schematic of artifact eccentricity is shown in Figure 2. The rotation axis of the aerostatic bearing was located at *O*_1_. The center of the artifact was *O*, which varied with the rotation angle. The measurement data obtained from the displacement sensor can be expressed as follows:

(3) $$d=d_{0}-d_{1}$$

where d is the measurement data; $d_{0}$ is the distance from rotation axis to sensor; and $d_{1}$ is the distance variable, which varies with rotation angle α and can be denoted as follows:

(4) $$d_{1}=e\;sin\;\alpha +\sqrt{r^{2}-{\left(l-e\;cos\;\alpha \right)}^{2}}$$

where e is the artifact eccentricity, r is the radius of the artifact, and l is the sensor position deviation from the spindle rotation axis. Figure 2b shows the sensor without position deviation, and Equation (4) can be rewritten as:

(5) $$d_{1}=e\;sin\;\alpha +\sqrt{r^{2}-{\left(e\;cos\;\alpha \right)}^{2}}$$

At a relatively small artifact eccentricity, the calculated result difference between the two equations can be ignored.

A single cycle of the rotational error data is a random signal, but multiple cycles are repeated signals. According to the Fourier theorem, the repetitive signals can be expressed as a sum of different-order harmonics. Based on the residual equation:

(6) $$\;\Delta d\left(\alpha_{i}\right)=d\left(\alpha_{i}\right)-\frac{1}{N}\sum_{i=0}^{N-1}d\left(\alpha_{i}\right)$$

Fourier coefficients can be expressed as

(7) $$a_{k}=\frac{2}{N}\left[\sum_{i=0}^{N-1}\Delta d\left(\alpha_{i}\right)\cdot cos\left(k\alpha_{i}\right)\right]$$

(8) $$b_{k}=\frac{2}{N}\left[\sum_{i=0}^{N-1}\Delta d\left(\alpha_{i}\right)\cdot sin\left(k\alpha_{i}\right)\right]$$

where *N* represents the measuring points, and *k* is the order of the harmonic. Therefore, in the polar coordinates, the rotation error can be expressed as

(9) $$\rho \left(\alpha \right)=r_{o}+\sum_{k=1}^{\infty }a_{k}\;cos\;k\alpha +\sum_{k=1}^{\infty }b_{k}\;sin\;k\alpha$$

where $r_{o}$ is the reference radius. The different harmonics of rotation error can be calculated by Equation (9). It is shown that the measuring accuracy of the rotation error can be significantly improved by improving artefact eccentricity. However, artifact eccentricity always existed in the measurement process. It is shown that the first harmonic was caused by artifact eccentricity. As indicated by Equation (10), eliminating the first harmonic in the measured data helps reduce the uncertainty resulting from artifact eccentricity.

(10) $$\rho \prime \left(\alpha \right)=\rho \left(\alpha \right)-\left[a_{1}\;cos\;\alpha +b_{1}\;sin\;\alpha \right]$$

## 3. Design and Development of the Measurement System

**Figure 3:**
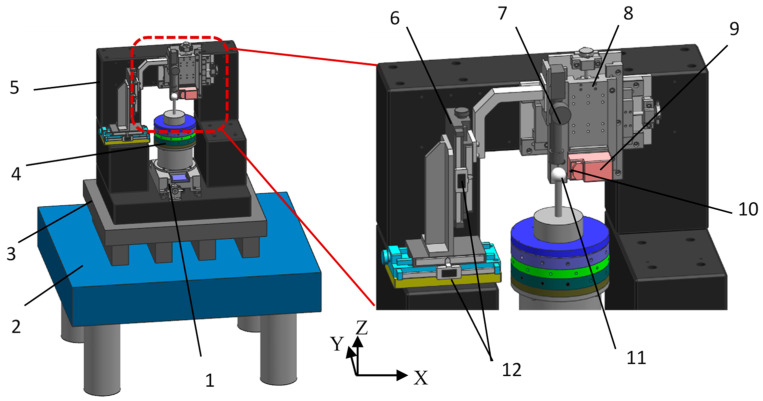
Design of the measurement system based on AFM: 1. guideway, 2. marble base, 3. active isolation platform, 4. aerostatic bearing, 5. marble frame, 6. adjusting module B, 7. optical microscope, 8. adjusting module A, 9. AFM scanning module, 10. atomic force tip, 11. artifact, 12. precision micrometer.

**Figure 4:**
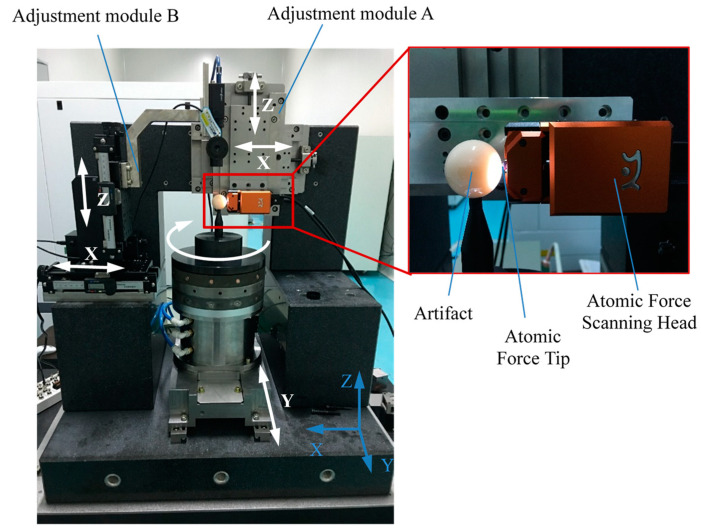
The developed measurement system for aerostatic bearing.

An AFM called NaniteAFM from Nanosurf Inc. (Lausanne, Switzerland) provides a solution for AFM integration with the least restriction to the sample dimensions. Therefore, in this paper, a subnanometer measuring system was designed and developed on the basis of the NaniteAFM, as shown in Figure 3, by using an active vibration isolating system and a marble base. Moreover, adjusting modules and AFM modules were fixed on the marble frame to reduce the deformation and influence of the vibration. The aerostatic bearing was vertically installed on the guideway, which could be moved in the *Y* direction with a resolution of 0.1 μm. Precision micrometers for recording the displacement variations were installed on the adjusting module B. The AFM module was installed on the adjusting module A, which could be moved in the *X* and *Z* directions with a resolution of 0.1 μm. A standard ball from Mahr Corp. (Rockland, MA, USA) with nominal roundness of 32 nm, was placed on the top of aerostatic bearing as an artifact. A probe installed on the atomic force scanning head was used to measure the displacement of the artifact during the rotation of the aerostatic bearing. Figure 4 shows the developed measurement system.

The eccentricity of the artifact should be regulated to be as small as possible to improve measurement accuracy. The optical microscope in this measurement system was used to adjust the eccentricity of the artifact. Based on the displacement recorded in precision micrometers, the eccentricity of the artifact can be regulated with a resolution of 0.2 μm before and after reversal. Because the probe was very fragile and the measurement distance of the AFM head was less than 6 μm, the microscope was necessary for monitoring the process of approaching the tip of the artifact. The adjusting module A was used to achieve the reversal of the AFM module; hence, the Donaldson reversal method can be applied to separate the artifact form error from the measurement results. Moreover, the aerostatic bearing was vertically installed on the guideway and, therefore, was isolated from the AFM module. Therefore, the effect of the microvibration of the aerostatic bearing on the measurement accuracy of the AFM module can be minimized or ignored.

The resolution of the AFM module had a significant influence on the measurement results of motion errors. In order to make the linearity of the AFM clear, calibration was performed using the standard nanometer step, as shown in Table 1. Table 2 shows the specifications of the AFM module used in the system. The precision of the AFM module mainly consists of tip precision and sampling precision. Using AFM to measure the motion accuracy of aerostatic bearing usually results in drastic tip wear. Therefore, the tip that was not coated must be characterized before and after the measurements. The tip was replaced by a new one when the difference between the two measurements was more than 20% to reduce the effects of tip wear on the measurement results.

## 4. Experimental Studies

**Figure 5:**
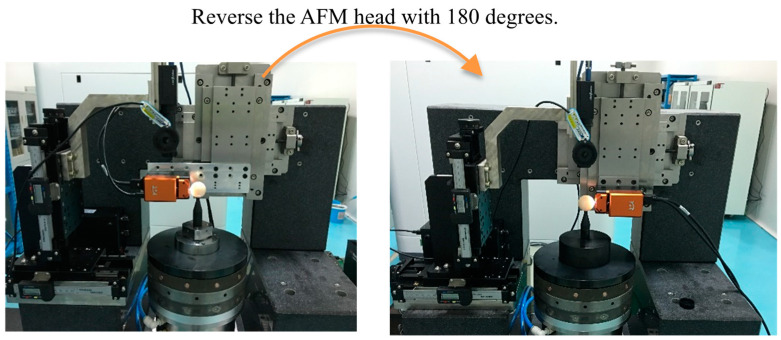
Measurement of motion error of aerostatic bearing based on Donaldson reversal method.

The measurement of motion errors of aerostatic bearing based on the Donaldson reversal method is shown in Figure 5. The aerostatic bearing was tested on the guideway of the measurement system. The temperature fluctuation was controlled within 0.1 °C to reduce the temperature drift. Moreover, the sensor positioning error can be controlled within 0.2° by the sensor fixture with ultra-high parallelism and flatness, and the measurement error can be reduced to a few percent of a nanometer, which can be ignored [14]. Before starting the measurement, the AFM displacement sensor and aerostatic bearing were allowed to operate for more than 1 h. Artifact eccentricity was limited to less than 0.2 μm.

**Figure 6:**
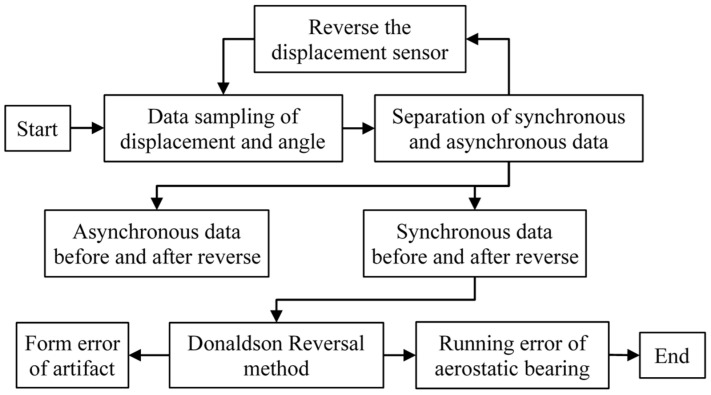
Flow chart of the measurement procedure.

**Figure 7:**
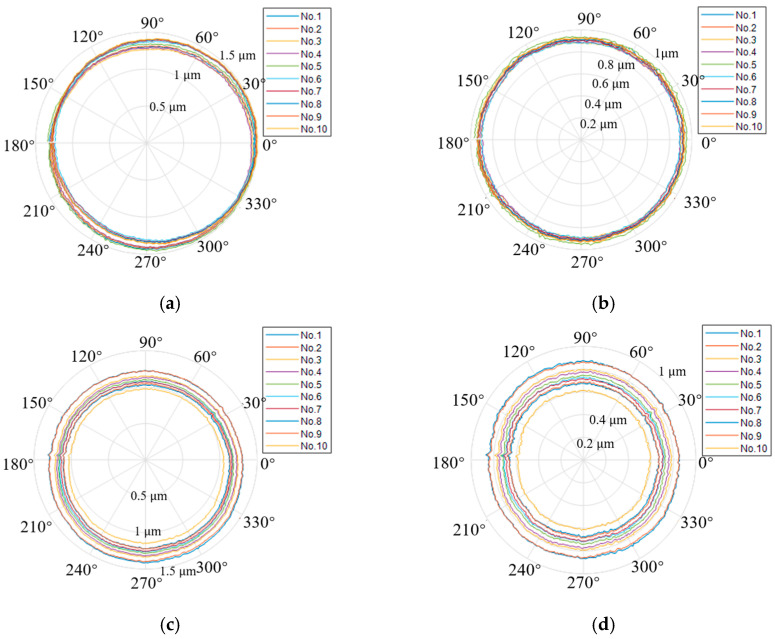
Measured results of motion errors: (**a**) original data and (**b**) data after eccentricity removal before reversal, and (**c**) original data and (**d**) data after eccentricity removal after reversal.

Figure 6 shows the flow chart of the measurement procedure based on the Donaldson reversal method. Displacement data and corresponding angles were sampled while synchronous and asynchronous data were separated. Then, the displacement sensor and artifact were reversed at 180°, followed by the separation of synchronous and asynchronous data. In this way, synchronous and asynchronous data before and after reversal were obtained. In total, ten tests for measuring the motion errors of an aerostatic journal bearing were undertaken, as shown in Figure 7.

**Figure 8:**
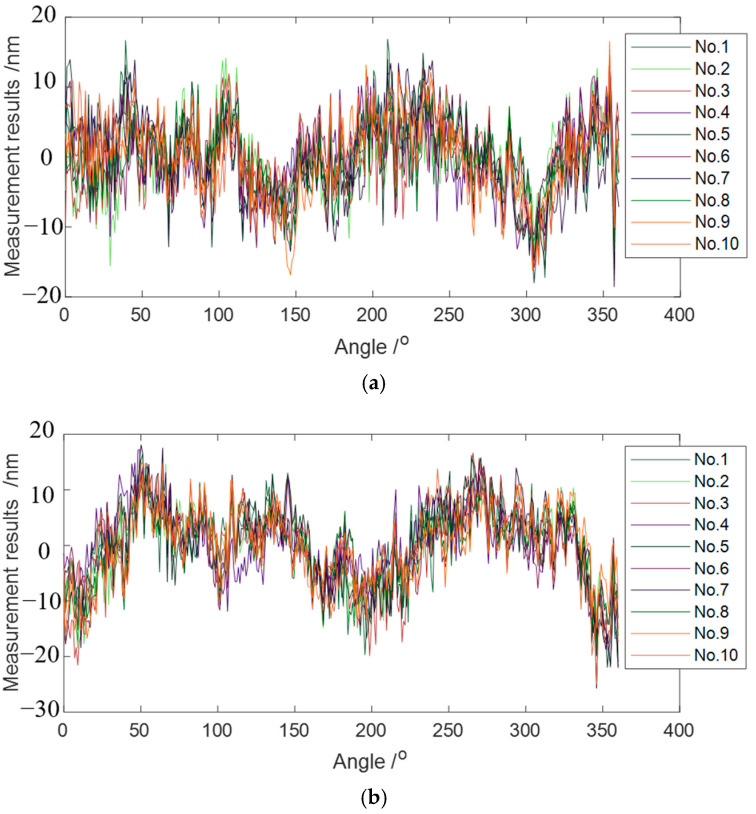
(**a**) Radial motion error of the aerostatic bearing and (**b**) form error of the artifact separated from the measured results.

The measured results before reverse were synchronous data combined with asynchronous data. The synchronous error can be obtained by averaging 10 groups of the combined consecutive measuring data, and the value was determined by calculating the peak-to-valley (PV) value of the 360 points in the averaging 10 groups of the combined data [15]. The asynchronous error value was obtained from the PV value of the aerostatic bearing at 100 revolutions, which took all 36,000 measuring points into account for the calculation [15]. The form error of the artifact and the motion error of the aerostatic journal bearing was calculated by Equation (2), as shown in Figure 8. Table 3 summarizes the measurement results. The measurement accuracy of the rotation error of the aerostatic bearing and the form error of the artifact were controlled within the subnanometer level. Moreover, the separated value of the artifact form error was very close to the nominal roundness of 32 nm.

## 5. Conclusions

In this paper, an ultra-high precision measurement system was proposed and developed to measure and characterize the running errors of aerostatic bearings based on the AFM scanning module. The Donaldson reversal method was used to separate the artifact form error from the measurement results, and the effect of artifact eccentricity was removed by eliminating the first harmonic of measured data. The effect of the microvibration of aerostatic bearings on the measurement accuracy of AFM was reduced or alleviated by the optimization design of the system structures. A series of experimental studies were undertaken, and the measurement results showed that the system with the integrated AFM module can achieve subnanometer precision for the separated results of the running error of aerostatic bearings and artifact form error, providing a new ultra-high precision method to measure the running accuracy of aerostatic bearings in the application of production engineering.

## Figures and Tables

**Table 1:** Calibration results of AFM.

Parameters	Performance Information	Measurement Data
Linearity of *X*	0.5%	0.31%
Linearity of *Y*	0.5%	0.26%
Noise of *X*	0.4 nm	0.3 nm
Noise of *Y*	0.4 nm	0.3 nm
Orthogonality of *X* and *Y*	1°	0.48°

**Table 2:** Specifications of the AFM module (NaniteAFM).

Items	Parameters
Scanning range (*X*, *Y*)	90 × 90 μm^2^
Scanning range (*Z*)	6 μm
Scanning frequency	1–2 Hz
Scan mode	Contact
Tip material	Silicon
Tip radius	5 nm
Measurement resolution	0.06 nm

**Table 3:** Experimental results of an aerostatic journal bearing.

No.	Synch. Error/nm	Asynch. Error/nm	Running Error/nm	Artifact Error/nm
1	48.3	4.4	39.1	32.1
2	48.2	3.8	39.3	31.8
3	48.2	4.6	39.2	32.0
4	48.2	4.1	39.3	31.9
5	48.2	4.0	39.2	32.0
6	48.3	3.9	39.2	32.1
7	48.2	3.6	39.3	32.2
8	48.2	4.4	39.4	32.0
9	48.1	3.9	39.3	31.9
10	48.2	4.0	39.3	32.1
Mean	48.2	4.07	39.26	32.01
Standard deviation	0.06	0.31	0.08	0.12

## Data Availability

Not applicable.
